# Electrospinning of antibacterial and anti-inflammatory Ag@hesperidin core-shell nanoparticles into nanofibers used for promoting infected wound healing

**DOI:** 10.1093/rb/rbac012

**Published:** 2022-02-18

**Authors:** Xiuli Ren, Yanan Hu, Linna Chang, Shibo Xu, Xifan Mei, Zhenhua Chen

**Affiliations:** Jinzhou Medical University, Jinzhou 121001, China

**Keywords:** antibacterial hydrogel, Ag nanospheres, wound healing, anti-inflammation

## Abstract

Bacterial infection and excessive inflammation are still the main obstacles to wound repair. Thus, antibacterial and anti-inflammation nanomaterials are always attracting for infected wound healing. In this work, ultra-uniform (∼20 nm) and colloidally stable Ag nanoparticles (Ag-Hes NPs) with core-shell structure were prepared by using hesperidin as reducing and capping agent. The obtained Ag-Hes NPs present effective antibacterial properties on both *Staphylococcus aureus* and *Escherichia coli*. Ag-Hes NPs also got high 1,1-diphenyl-1-picrylhydrazyl scavenging capability of 69%. Under the package of polyvinyl alcohol and sodium alginate, Ag-Hes NPs were encapsulated into electro spun nanofibers to form hydrogel (Ag-Hes@H). This strategy provides a moisture environment which could enrich and release Ag-Hes NPs gradually. Cell experiments and animal wound healing investigation proved that Ag-Hes@H could promote the proliferation and migration of human umbilical vein endothelial cells and accelerate infected wound healing. Meanwhile, Ag-Hes@H significantly reduced the expression of inflammatory cytokines, including IL-6, MMP9 and TNF-α. Immunohistochemistry data further suggested that Ag-Hes@H accelerated wound closure by promoting collagen deposition and skin cell proliferation. The designed antibacterial and anti-inflammatory Ag-Hes@H has great potential for promoting infected wound healing.

## Graphical Abstract

**Figure:**
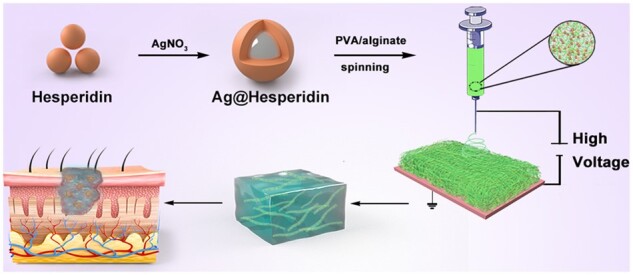

## Introduction

The treatment of chronic wounds infected by bacteria is still a thorny problem in clinical field, and a severe threaten to human health [1, 2]. The invasion of bacteria during wounds repair, especially in the case of antibacterial membrane and drug-resistant bacteria, is extremely difficult to treat with traditional antibiotics. To make matters worse, new antibiotics are increasingly difficult to develop [3–5]. Bacterial infection and excessive inflammation are still the main challenges for infected wound repair. Therefore, antibacterial and anti-inflammatory nanomaterials are always attracting for infected wound treatment [6–9]. Compared with these antibiotics, nanomaterials have attracted extensive attention, such as gold [10–12], silver [13–15] and copper nanoparticles [16–18].

Among them, silver nanoparticles, due to their high surface volume ratio and unique broad-spectrum antibacterial ability, have been widely studied [19–21]. However, Ag^+^ and Ag NPs are potentially toxic to mammalian cells [22]. Meanwhile, without modification, bare Ag NPs tend to aggregate, which will reduce their antibacterial efficiency and hinder their application [23, 24]. To overcome these shortages, we propose a strategy of designing colloidally stable Ag nanoparticles by using hesperidin as reducing and capping agent, which could avoid aggregation to gain lower toxicity and higher antibacterial efficiency.

**Figure 1.**
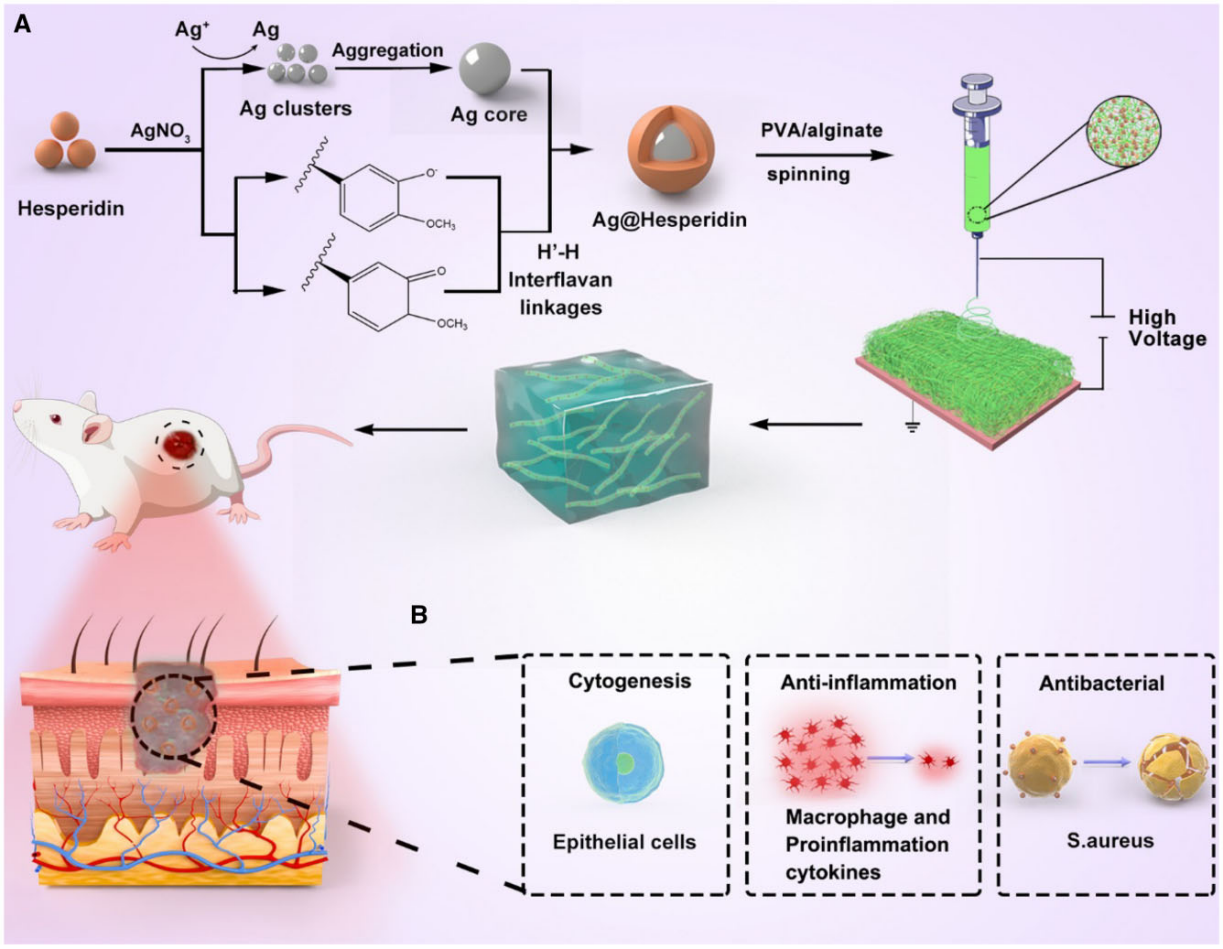
(**A**) Schematic preparation procedure of Ag-Hes NPs, Ag-Hes NPs electro spun nanofibers and Ag-Hes@H; (**B**) possible mechanisms of Ag-Hes@H in promoting infected wound healing in animal experiment

Hesperidin is a kind of flavonoid with antibacterial and antioxidant properties, which are beneficial for promoting wounds healing [25–28]. Herein, in this work, hesperidin was used as reducing and capping agent to prepare colloidally stable Ag nanoparticles (Ag-Hes NPs). The preparation procedure of Ag-Hes NPs is shown in the Fig. 1A. Briefly, silver ions are reduced by hesperidin to form Ag nanoclusters, then, the aggregated clusters grew up to form the Ag core. Meanwhile, hesperidin molecules were oxidized and polymerized to form the shell of Ag core through interflavan linkages reported in our previous work [29]. In this process, hesperidin was used as both reducing and capping agent. Ag ions were reduced by Hes to form Ag clusters, then, these clusters aggregated into Ag nanoparticles. Meanwhile, Hes molecules were oxidized into structures similar to quinone and semi-quinone. Then, the oxidized Hes structures could polymerized into shell to encapsulate the Ag nanoparticles to form Ag@Hesperidin nanoparticles (Ag-Hes NPs). Subsequently, after centrifugal collection and cleaning, the obtained Ag-Hes NPs were packaged by polyvinyl alcohol (PVA) and sodium alginate (Alg) using the strategy as previously reported [30–32]. The above mixture was prepared into electro spun nanofibers to form hydrogel (Ag-Hes@H). Hydrogels and nanofibrous materials are very attracting in medical application materials field [33–37]. Finally, the designed Ag-Hes@H was expected to have great potential for promoting infected wound healing in animal experiment. As illustrated in Fig. 1B, Ag-Hes@H may play multiple functional roles of antibacterial, anti-inflammation and promoting the proliferation and migration of skin cells.

## Materials and methods

### Materials

Silver nitrate (AgNO_3_, 99%) and hesperidin were all obtained from Sigma-Aldrich (St. Louis, MO). Methicillin-resistant *Staphylococcus aureus* and Gram-negative *Escherichiacoli* were purchased from the Guangdong Microbial Culture Preservation Center (China). Dulbecco modified Eagle medium, 3-(4,5-dimethylthiazol-2-yl)-2,5-diphenyl tetrazolium bromide (MTT) and fetal bovine serum (FBS) were derived from GIBCO (USA). American Type Culture Collection provided human umbilical vein endothelial cells (HUVECs); 1-methyl-2-pyrrolidone was purchased from Aladdin. Anti-CD31, vascular endothelial growth factor (VEGF), basic fibroblast growth factor (bFGF) and tubulin were acquired from Cell Signaling Technology (USA).

### Preparation of the Ag-Hes and Ag-Hes@H

AgNO_3_ (2 mL, 3.397 mg/mL) was stirred at room temperature for 30 min, then, 10 mL hesperidin solution (17.6 mg/mL) was reacted at room temperature for 1 h. After that, the obtained Ag-Hes NPs were centrifugal collected and rinsed. The obtained samples were combined within PVA-Alg for electrospinning. The electrospinning experiments were carried out at ambient temperature. The hybrid solution was added into a 5 mL syringe with a needle (inner diameter of 0.4 mm). A clamp connected with high voltage power supplier (providing positive voltage from 0 to 50 kV) was attached on the needle. A piece of aluminum foil was placed below the needle at the distance of 15 cm as grounded collector. The polymer jets generated from the needle by high voltage flied to the grounded collector and formed the nanofibers. The applied voltage and flow rate of the solution were fixed at 18 kV and 0.3 mL/h, respectively. The obtained electro spun nanofibers were collected to form nano hydrogels (Ag-Hes@H) using the strategy as previously reported [30–32]. Meanwhile, the Ag NPs were prepared by using NaBH_4_ reduced method [38].

### Characterization

The morphology of Ag and Ag-Hes NPs was analyzed by transmission electron microscopy (TEM, JEM-1200EX, Tokyo, Japan). The size of Ag-Hes nanoparticles was characterized by dynamic laser scattering (DLS, Malvern, NanoZS90, Worcestershire, UK). UV-vis data were obtained by UV-vis spectrophotometer (PerkinElmer Lambda 605S UV-vis spectrometer). Fluorescence data were obtained using a fluorescence photometer (F97PRO, Shanghai, China). The component of Ag-Hes nanoparticles was analyzed by Fourier transform infrared spectroscopy (FTIR, SHIMADZU, Kyoto, Japan) with the KBr disk method and powder X-ray diffraction analysis (XRD, Shimadzu, Kyoto, Japan) with Cu K radiation. The thermogravimetric (TG) analyzer (METTLER TOLEDO, TGA/DSC1/1100, Switzerland) measured the variation of the sample in the temperature range from 50°C to 800°C, using an empty aluminum plate as a reference. Confocal laser scanning microscopy (CLSM, Leica TSCSP5 confocal unit) was used to observe the expression of related proteins in cells. The apoptosis-related proteins were detected by western blot.

### Antibacterial activity

The turbidity method was used to assess the antibacterial activity of Ag-Hes. Specifically, glassware was autoclaved for 30 min before experiments. Then, *S. aureus* suspensions (10^8^ CFU/mL) with Ag NPs or Ag-Hes NPs were incubated for 5.5 h at 37°C (300 r/min). The control group did not add materials. Finally, photos were taken to record the change of turbidity.

The filter paper diffusion technology was further used to demonstrate the antibacterial properties of Ag-Hes against *S. aureus*. First, the *S. aureus* suspensions (10^8^ CFU/mL) were uniformly coated on the agar plate. Subsequently, the filter sheet containing Ag NPs and Ag-Hes NPs was spread on *S. aureus* coated agar plates, respectively. The diameter of the bacteriostatic ring in each group was observed after 24 h of incubation. The survival rate of *S. aureus* was also assessed. The *S. aureus* suspensions (10^8^ CFU/mL) were mixed with Ag NPs and Ag-Hes NPs in a 1.5 mL tube for 30 min, respectively. Then, we continued to incubate them on the LB Agar Medium for 1 day. Finally, the survival rate was calculated.

### Antioxidant capacity of Ag-Hes@H

The antioxidant capacity of the Ag-Hes@H was determined by 1,1-diphenyl-1-picrylhydrazyl (DPPH) assays. The DPPH radical scavenging assay was performed according to the Blois method. DPPH radical solution (1.0 mM) in ethanol was prepared. Then, this solution was mixed with Ag-Hes@H to give final antioxidant concentrations of 30, 60, 90 or 120 µM. Finally, after 30 min, the absorbance was measured at 517 nm. The percentage of DPPH radical scavenging was calculated. All experiments were performed three times and the average values were calculated.

### Cell viability assay

The proliferation of HUVECs was measured by MTT assay at different time. First, HUVECs (5000 cells/holes) were cultured in 96-well plates for 24 h. Then, HUVECs (5000 cells/holes) were starved overnight on a medium containing no FBS. At 6, 12 and 24 h, 20 μL MTT solution (5 mg/mL in FBS) was added into each hole and incubated for 4 h. After discarding the night, 150 μL dimethyl sulfoxide was added into each hole and incubated for 15 min in the dark. Finally, the quantitative detection was carried out on the 490 nm enzyme-labeled instrument.

### *In vitro* migration experiments

The migration capability was reflected by Scratch assay. HUVECs (8 × 10^4^/wells) were seeded in a 12-well plate. We then drew a uniform line with the sterile fluid suction head after the cell reached 80% of the plate, scratching the cells at the bottom of the plate. The debris was washed with PBS. The treatment group was then treated with NH, Ag@H and Ag-Hes@H, respectively. The treatment group was then treated with NH, Ag@H and Ag-Hes@H in the incubator at 37°C, respectively. Fragments were washed with PBS. Then, the cells were treated with 4% paraformaldehyde, incubated with 0.1% Triton X-100 and then stained with 4′,6-diamidino-2-phenylindolesolution. Through an inverted microscope, 0 h, 12 h, images were obtained 24 h. The migration ability was tested by the ratio of closed area to initial wound migration area (%) = (initial wound area – residual wound area)/initial wound area × 100%.

### Animal wound healing investigation

In order to evaluate the therapeutic effect of Ag-Hes@H on wound healing, infected wound healing animal model was established. The selected SD rats were randomly divided into four groups, and each group included five rats. All the rats were anesthetized and shaved, and the wounds of about 1.76 cm^2^ were incised on the back and injected with 200 μL *S.aureus* solution (10^8^ CFU/mL). After continuous infection for 3 days, pure hydrogels, Ag@H, and Ag-Hes@H were applied to the wounds of rats in each group, and a blank control group was set. After 12 days, the rats in each group were sacrificed and the wound tissue was extracted. Animal studies were conducted in accordance with the guidelines of the Institutional Animal Care and Use Committee of Jinzhou Medical University.

### Histology analyses

For histological analysis, wound skin sections were collected on Day 12 after treatment. The wound tissue from each group was fixed in neutral buffered formalin. After routine sectioning, two manipulations, hematoxylin and eosin (H&E) staining and Masson staining, these samples were used to evaluate the wound healing status of each group. Sections were photographed with a Leica DM4000B microscope.

### Cellular immunofluorescence test

The HUVECs were seeded in confocal cell dishes and incubated for 24 h. The cells were washed three times with phosphate buffer. Then, we used 4% paraformaldehyde to immobilize cells for 40 min. Subsequently, the cells were treated with 0.1% Triton X-100. Then, they were blocked with normal goat serum for 60 min. The HUVECs were incubated with anti-bFGF and anti-tubulin overnight at 4°C. The second antibody was added into the goat serum and cultivated for 2 h. The cells were washed three times and stained with DAPI for 15 min. Finally, CLSM was used to observe the staining cells.

### Western blot

The expression of correlated proteins was estimated by western blot. First, cells or tissues were collected around the wound. Then, the samples were separated by polyacrylamide gel electrophoresis, transferred from the polyacrylamide gel to the polyvinylidene fluoride film. They were closed at room temperature by 5% milk for 2 h. After that, the corresponding primary antibodies included anti-rabbit bFGF, Tubulin, silent information regulator 1 (Sirt 1), NF-κB, MMP9, TNF-α were added for 2 h. Protein bands were visualized with the BIO-RAD imaging system (Bio-Rad, USA). The strength of the bands was analyzed using Image J software.

### Statistical analysis

All independent experiments were carried out under corresponding conditions. The test and analysis of statistical significance adopt the one-way analysis of variance and analysis of variance. *P < 0.05, ** *P* < 0.01 and *** *P* < 0.001 are statistically significant.

## Results and discussion

**Figure 2.**
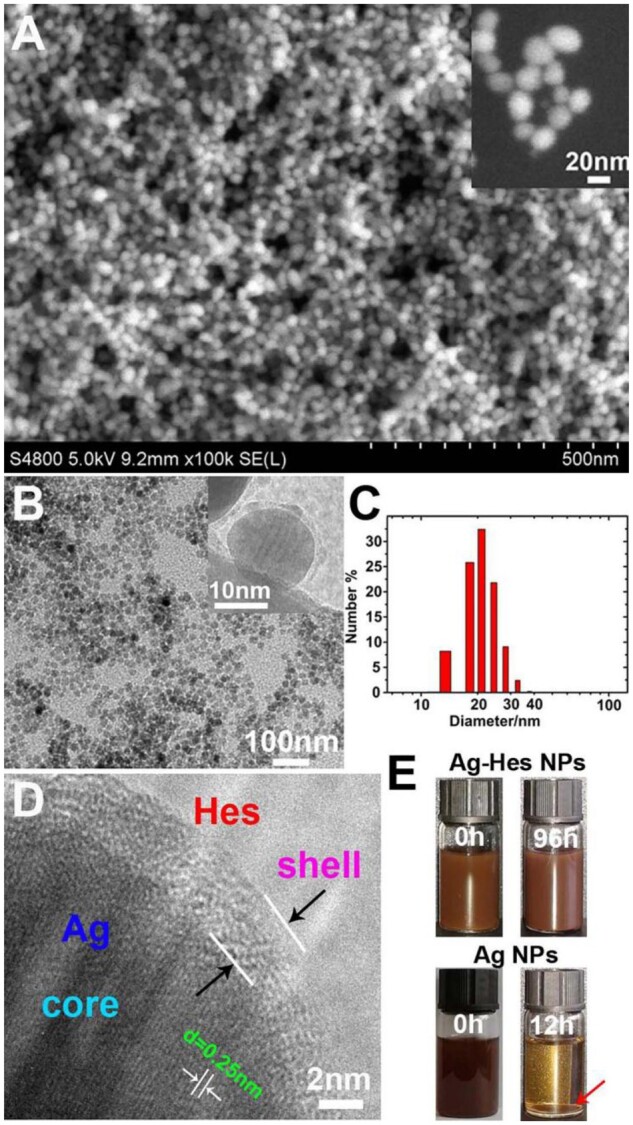
(**A**) SEM image of Ag-Hes NPs, the insert was magnified SEM image of Ag-Hes NPs; (**B**)TEM image, the insert was TEM image of a single magnified Ag-Hes nanoparticle; (**C**) DLS size distribution of Ag-Hes NPs; (**D**) magnified HRTEM image of one Ag-Hes nanoparticle; (**E**) settlement experiment photos of Ag and Ag-Hes nanoparticles in water

Supplementary Figure S1 showed the spatial structure of the Hes molecule. The abundant hydroxyl functional groups make it a high efficiency crystal growth modifier [39]. Morphology of the synthesized Ag-Hes was characterized the by SEM and TEM (Fig. 2). As showed in Fig. 2A, compared with the agglomerated pure Ag particles (Supplementary Fig. S2, without Hes as modifier), the prepared Ag-Hes NPs were ultra-uniform (∼20 nm in diameter) and colloidally stable. TEM image (Fig. 2B) confirmed the uniform shape and good dispersion of the obtained Ag-Hes NPs. The insert in Fig. 2B further indicated the core-shell structure of Ag-Hes NPs. DLS result (Fig. 2C) further confirmed the narrow size distribution of Ag-Hes was around 20 nm. HRTEM image in Fig. 2D clearly indicated the Ag core was encapsulated by a layer of 2 ∼ 3 nm Hes shell. The lattice spacing of 0.25 nm for Ag core could also be discriminated in Fig. 2D, which corresponds to the {111} reflection [20]. Settlement experiment photos (Fig. 2E) of Ag and Ag-Hes NPs in water revealed that Ag-Hes NPs could stably disperse in water for 96 h, while the nude Ag nanoparticles has precipitated completely only for 12 h. These results clearly proved the significant role of Hes in affording the good stability of Ag-Hes in an aqueous solution.

**Figure 3.**
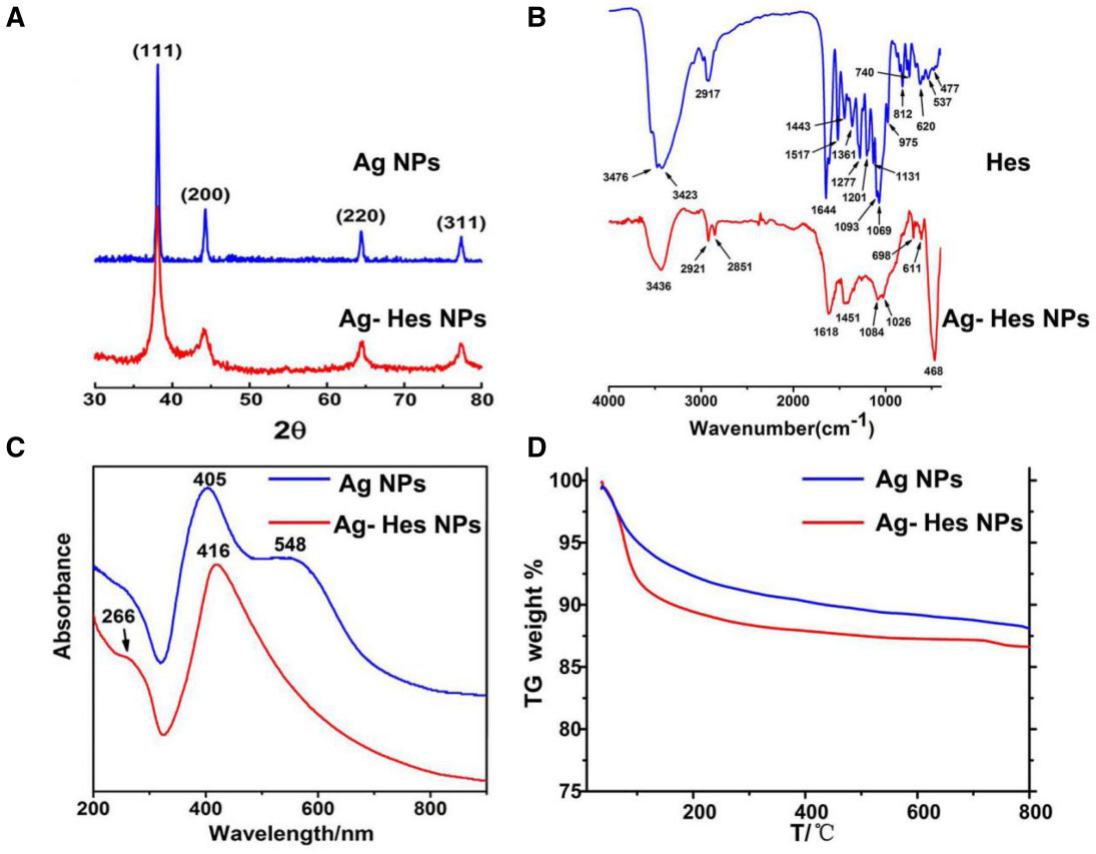
(**A**) XRD patterns; (**B**) FTIR spectra; (**C**) UV–vis; (**D**) TG curves of the samples

The XRD patterns (Fig. 3A) confirmed the crystal structure of Ag-Hes. The diffraction peak of the Ag-Hes NPs is consistent with that of the Ag NPs, the diffraction peaks at 38.1°, 44.2°, 64.5° and 77.5°, respectively, corresponding to {111}, {200}, {220} and {311} diffractions of Ag (JCPDS 04-0783). Peaks of Ag-Hes are broader than those of Ag group might be because the smaller size and the modification of HES molecules would make the XRD peaks wider. FTIR spectra (Fig. 3B) further confirmed the incorporation of Hes in Ag-Hes. The characteristic peaks of Hes were observed in the FTIR spectra of Ag-Hes, absorption peak at 3426 cm^−1^ was the O–H stretching vibration of Hes, while the peak at 1618 cm^−1^ referred to the stretching vibrations of C=C or C=O groups of Hes. Absorptions at 1084 and 1026 cm^−1^ were attributed to the C–O stretch of Hes. Figure 3C present the UV-vis spectra of the prepared Ag-Hes and pure Ag NPs. UV spectrum of Ag NPs showed a characteristic absorption peak at 405 nm, and a shoulder peak at 548 nm, which might suggest that they have bulk or aggregated silver particles (confirmed by Fig. S2) in the samples [40]. Hes showed a characteristic absorption peak at 286 nm (Supplementary Fig. S3). Ag-Hes displayed two absorption peaks at 266 and 416 nm, which are attributed to the characteristic absorption peak of Hes and the plasma resonance of Ag NPs, respectively. The shift of maximum absorbance wavelength of Ag-Hes may attribute to the phenomenon of surface plasmon resonance absorption band. In addition, no shoulder peak at 548 nm on Ag-Hes UV spectrum suggests that the Ag-Hes NPs were uniform and no aggregated bulk particles, which was consistent with the SEM, TEM and DLS results in Fig. 2. Besides the above analysis, to determine the amount of Hes in Ag-Hes, TG analysis was used to analyze the composition of Hes in Ag-Hes NPs. As shown in Fig. 3D, Ag-Hes had a greater weight loss than pure Ag NPs during combustion process. The TG result indicted that the amount of Hes incorporated in Ag-Hes NPs was 1.7 wt%.

**Figure 4.**
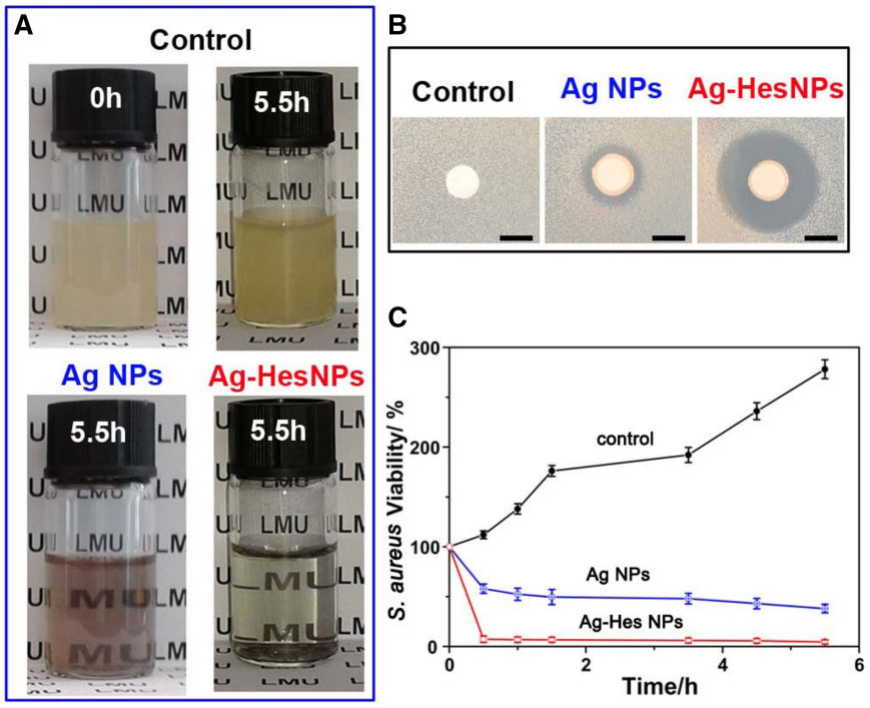
(**A**) Photos of *S. aureus* cultured in different materials at 0 and 5.5 h; (**B**) zone of inhibition (DIZ) experiment results of the different materials against *S. aureus*; (**C**) time-dependent viability curves of *S. aureus* with different samples

After thorough morphology and composition characterization, the antibacterial property of the obtained Ag-Hes NPs was investigated. Figure 4 provided the antibacterial activity of Ag-Hes. Figure 4A showed photos of *S. aureus* cultured in different materials at 0 h and 5.5 h. Compared with the *S. aureus* cultured in the control group, the addition of Ag NPs into the culture system could effectively reduce the turbidity of *S. aureus* solution, which proved the obvious antibacterial effect of the added Ag NPs. While for Ag-Hes, the turbidity of the culture system was significantly reduced than that of Ag NPs group. The excellent antibacterial property of Ag-Hes was further validated by the zone of inhibition experiment, the results are presented in Fig. 4B. No obvious inhibition circle was observed in the control group, in contrast, the other two groups showed obvious inhibition rings. In addition, the diameter of the antibacterial ring in the Ag-Hes NPs group was significantly broader than that of Ag NPs group. This contrast again proved that the antibacterial activity of the Ag-Hes NPs group was better than that of Ag NPs. To further investigate the antibacterial performance of Ag-Hes, antibacterial kinetic curves for *S. aureus* (Fig. 4C) and *E.coli* (Supplementary Fig. S4) were performed. Unlike the increased trend of *S. aureus* viability in control group, Fig. 4C revealed that the viability of *S. aureus* in Ag NPs and Ag-Hes NPs groups decreased obviously. Especially, after cultured for 5 h, it was worth noting that the inhibition rate of Ag-Hes NPs was 94.5%, which was much lower that of 62% for Ag NPs. The reason of Ag-Hes NPs having better antibacterial activity than Ag NPs might attributed to two aspects brought by Hes molecules. The first aspect is that Hes molecules have efficiently improved the uniformity of the prepared nanoparticles as illustrated in Fig. 2 and Supplementary Fig. S2. It is known that agglomeration will significantly reduce the antibacterial ability of Ag nanoparticles. The second aspect is that the antioxidant activity of Hes helps Ag-Hes NPs destroy bacterial biofilm, which is beneficial to get better antibacterial capability. The antioxidant activity Ag-Hes was evaluated by DPPH tests. As shown in Supplementary Fig. S5, the DPPH scavenging rate increased significantly with the increase of Ag-Hes concentration, and finally reached 69%.

In addition to good antibacterial properties, the biocompatibility of Ag-Hes NPs is also a key factor affecting its biomedical applications. Therefore, HUVECs cell viability incubated under different conditions was investigated. As shown in Supplementary Fig. S6, Ag NPs present obvious toxicity to HUVECs; however, Ag NPs did not show obvious toxicity to the cells. This might contribute by the encapsulation of Hes molecules which lower the toxicity of Ag.

**Figure 5.**
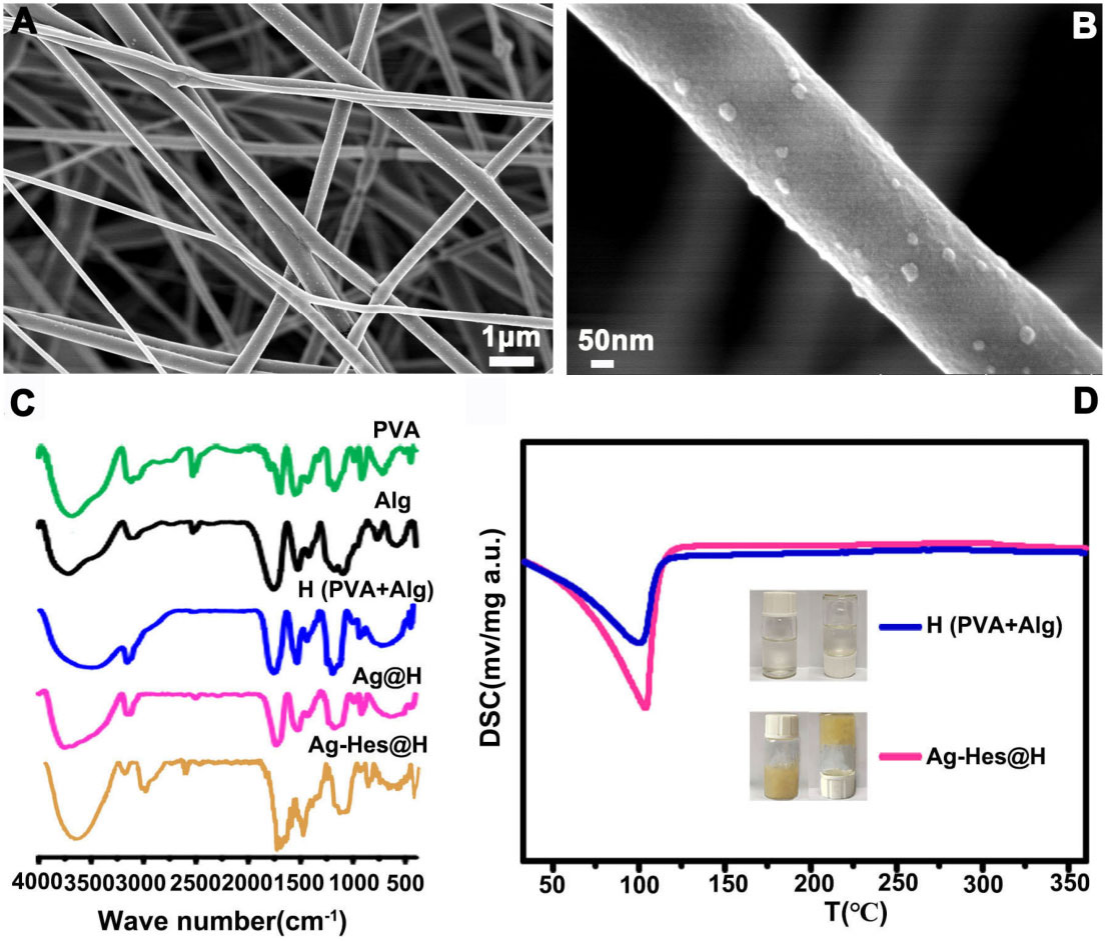
(**A**) SEM image of Ag-Hes NPs electro spun nanofibers; (**B**) magnified SEM image single Ag-Hes NPs electro spun nanofiber; (**C**) FT-IR spectra of the samples; (**D**) DSC of pure PVA-Alg hydrogel and Ag-Hes@H

Based on the detailed characterization and analysis of the morphology, composition, antibacterial properties and biocompatibility of Ag-Hes NPs, the preparation of Ag-Hes electro spun nanofibers and hydrogel was carried out in order to apply it to animal infected wound healing. Figure 5A provided the SEM image of the obtained electro spun nanofibers. It could be seen from Fig. 5A that nanoparticles were attached to these nanofibers. Figure 5B further clearly showed these Ag-Hes NPs were decorated on the prepared nanofibers. After gelled by these nanofibers, the prepared hydrogel samples were characterized by FT-IR and differential scanning calorimetry (DSC). In Fig. 5C, the broad absorption band at 3620 cm^−1^ was the absorption peak of –OH; the absorption at 2917 cm^−1^ was the C–H stretching vibration peak in hesperidin and the peak at the 1644 cm^−1^ was the characteristic absorption peak of C = O. Figure 5D showed the DSC curves of the samples, the exothermic peak of water for Ag-Hes@H was at a higher temperature than that of pure PVA-Alg hydrogel. This suggests Ag-Hes@H has stronger moisture retention performance than the pure hydrogel, which might due to the stronger hydrogen bond interaction between water and Ag-Hes@H’s building blocks. The DSC difference between Ag-Hes@H and H might also be associated with the increased viscosity, which was further proved by the insert photos in Fig. 5D. The inserted photos in Fig. 5D also prove that Ag-Hes@H has a higher viscosity than that of H. This might because the addition of Ag-Hes nanoparticles may enhance the hydrogen bonds in the PVA-Alg gel network through the abundant hydroxyl groups on the Hes molecules. Meanwhile, Ag has formed a large number of metal functional groups complexation with hydroxyl and carboxyl groups on PVA and Alg. These effects make Ag-Hes@H have higher viscosity than H. This is consistent with our previous works [30, 32].

**Figure 6.**
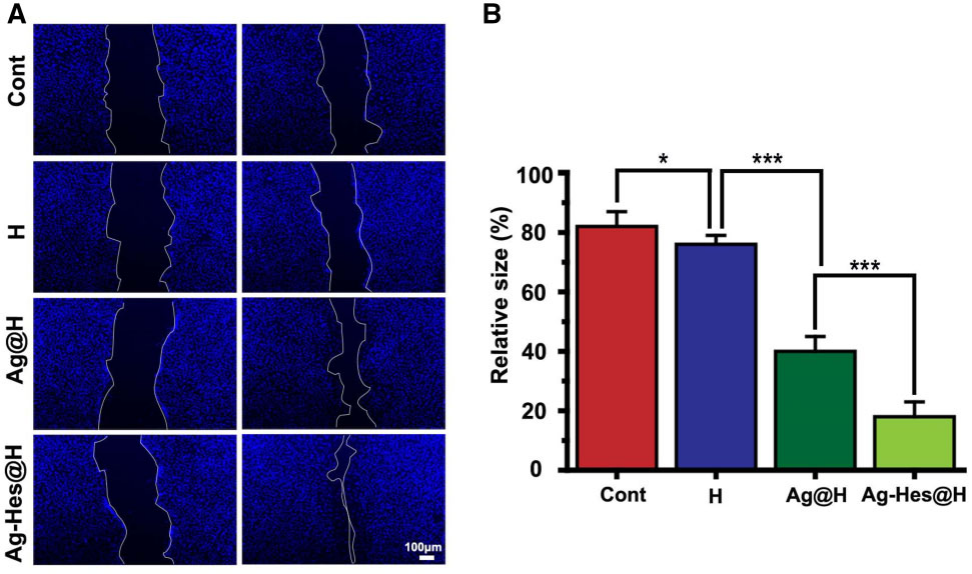
(**A**) The micrographs of the scratch-wound assay in different groups, blue represents HUVECs nucleus (scale bar for all images is 100 μm); (**B**) quantification of HUVEC migration rates analysis, *P < 0.05, **P < 0.01 and ***P < 0.001

Endothelial cell migration is critical for angiogenesis, therefore, the regenerative ability of Ag-Hes@H was further investigated through HUVECs. Under different treatments of cells scratch, the migration extent revealed difference (Fig. 6A). Compared with the control group with no significant cell migration, the scratch gap of the treatment group diminished in varying degrees. The scratch gap in the Ag-Hes@H group after 12 h incubation became significantly smaller compared with the other two treatment group, which means that Ag-Hes@H could noticeably enhanced the migratory ability of HUVECs cells. The quantitative analysis results were reported in Fig. 6B, the relative size of scratch area in the Ag-Hes@H group was < 20%, which was significantly smaller than that in other groups, indicating that the Ag-Hes@H group had higher cell migration rate.

**Figure 7.**
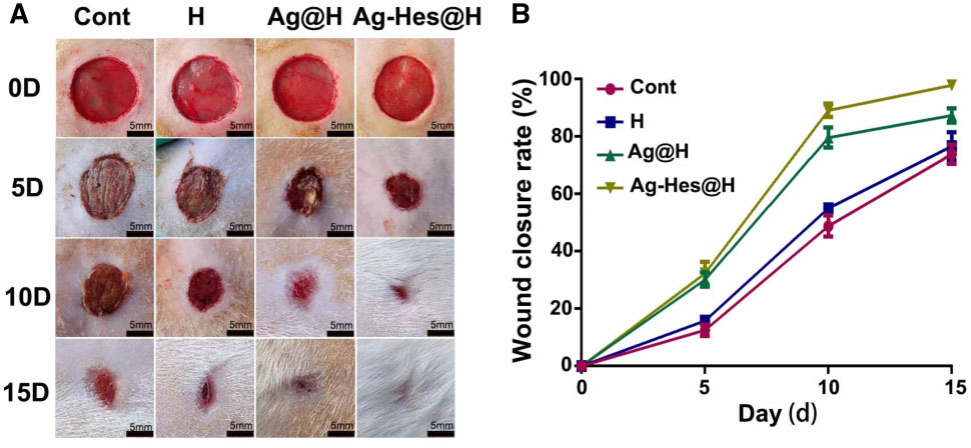
Evaluation effects of accelerating infected wound *in vivo*. (**A**) Images of infected wound healing at different times, bars are all 5 mm; (**B**) corresponding statistical graph of relative wound area from each group with different treatments

The repair effect of Ag-Hes@H on male rat wound infected by methicillin-resistant *S.aureus* has been investigated. The digital photographs of the wounds and quantification of closure rates at the 5th, 10th and 15th post-wounding days (Fig. 7A) showed that Ag-Hes@H significantly accelerated the closure of the rat wound, after 15 days, the wound healing closure rate was close to 97%. Compared with the rat in the blank control group and the pure hydrogel group, the Ag@H-treated group also showed a good wound closure rate, and the wound healing rate reached about 83% (Fig. 7B). However, its closure rate was still much lower than that of Ag-Hes@H-treated group. This might suggest that bacterial inhibition plays significant role in promoting wound closure.

**Figure 8.**
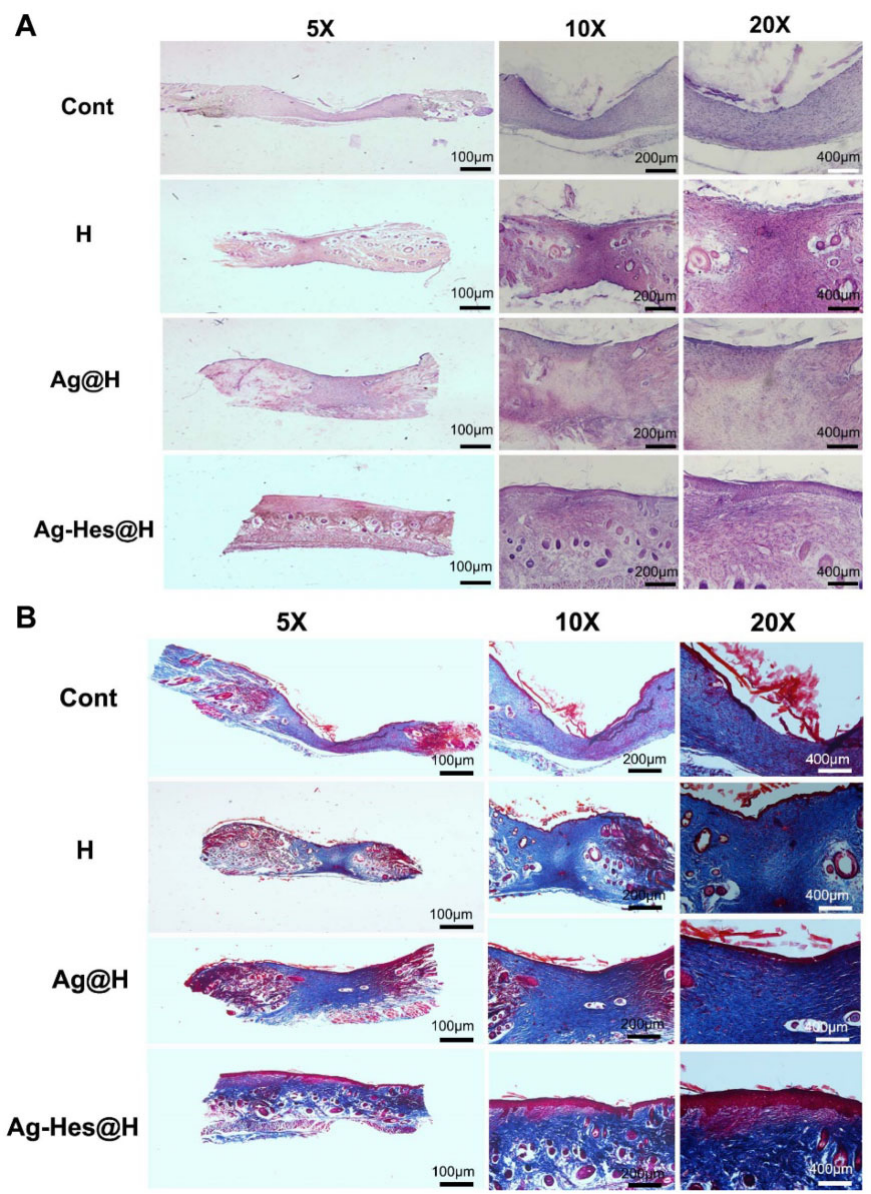
(**A**) H&E staining of wound sites of control group and the group with the treatment of H, Ag@H and Ag-Hes@H, respectively, on the 10th day after surgery; (**B**) Masson staining of wound sites with the treatment of NH, Ag@H and Ag-Hes@H, respectively, on the 10th day after surgery; scale bars are all 100 μm

Immunohistochemistry changes of wound healing in different groups were collected by using H&E and Masson staining methods. In order to further investigate the information of regenerative skin of the wound, the wound samples of rats on the 12th day after injury were collected and treated for further analysis. Figure 8A showed that epithelialization in the control group and H group was not as complete as those of the Ag@H group and Ag-Hes@H group. It is obvious that Ag-Hes@H group presents the best re-epithelialization process. Figure 8B revealed that the collagen fiber bundles in control group and H group were few, loose and disordered. But, Ag@H and Ag-Hes@H groups had many collagenous fibers, arranged in an orderly fashion, and blood vessels and hair follicles began to proliferate. In particular, the collagen fibers in the Ag-Hes@H group were strongest, and the collagen deposition and the proliferation of skin cells at the wound surface were significantly increased.

**Figure 9.**
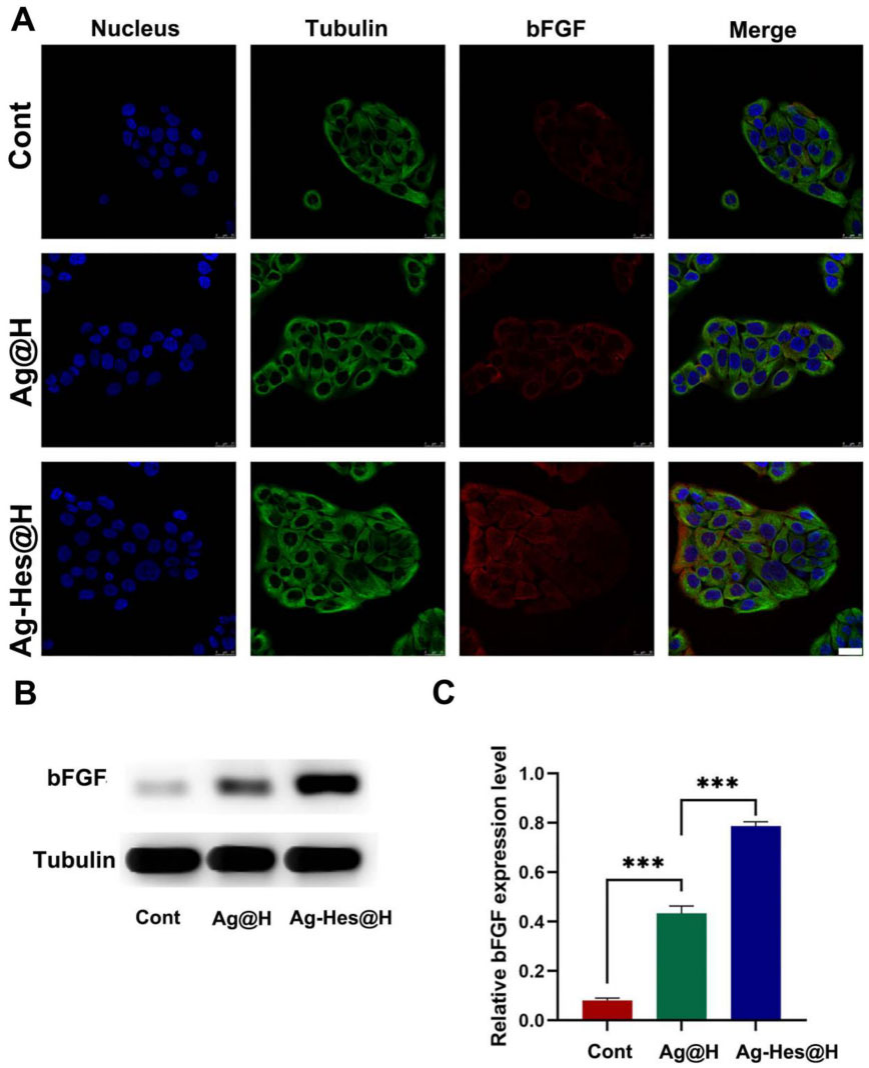
(**A**) Confocal laser scanning microscopy (CLSM) images of the expression analysis of bFGF after different treatments, bar = 25 μm; (**B**) the expression level of bFGF protein analyzed by western blot; (**C**) quantification analysis of the bFGF, ***P < 0.001

To further elucidate the ability of Ag-Hes@H to accelerate epithelial, the expression of bFGF was tested in cell experiment cultured under various conditions. bFGF is known to accelerate skin regeneration in wound therapy. As presented in Fig. 9A, the weak red fluorescence in the control group means low bFGF expression. On the contrary, the increased red fluorescence of bFGF were observed from Ag@H to Ag-Hes@H group. The strongest red fluorescence appeared in Ag-Hes@H group meant that the expression of bFGF was markedly enhanced by Ag-Hes@H. To further confirm this trend, the western blot assay and quantitative analysis of bFGF protein were adopt, the results were present in Fig. 9B and C, respectively. Compared with the other two groups, the expression of bFGF in Ag-Hes@H group was marked up-regulated, which was consistent with the previous animal experiment results.

**Figure 10:**
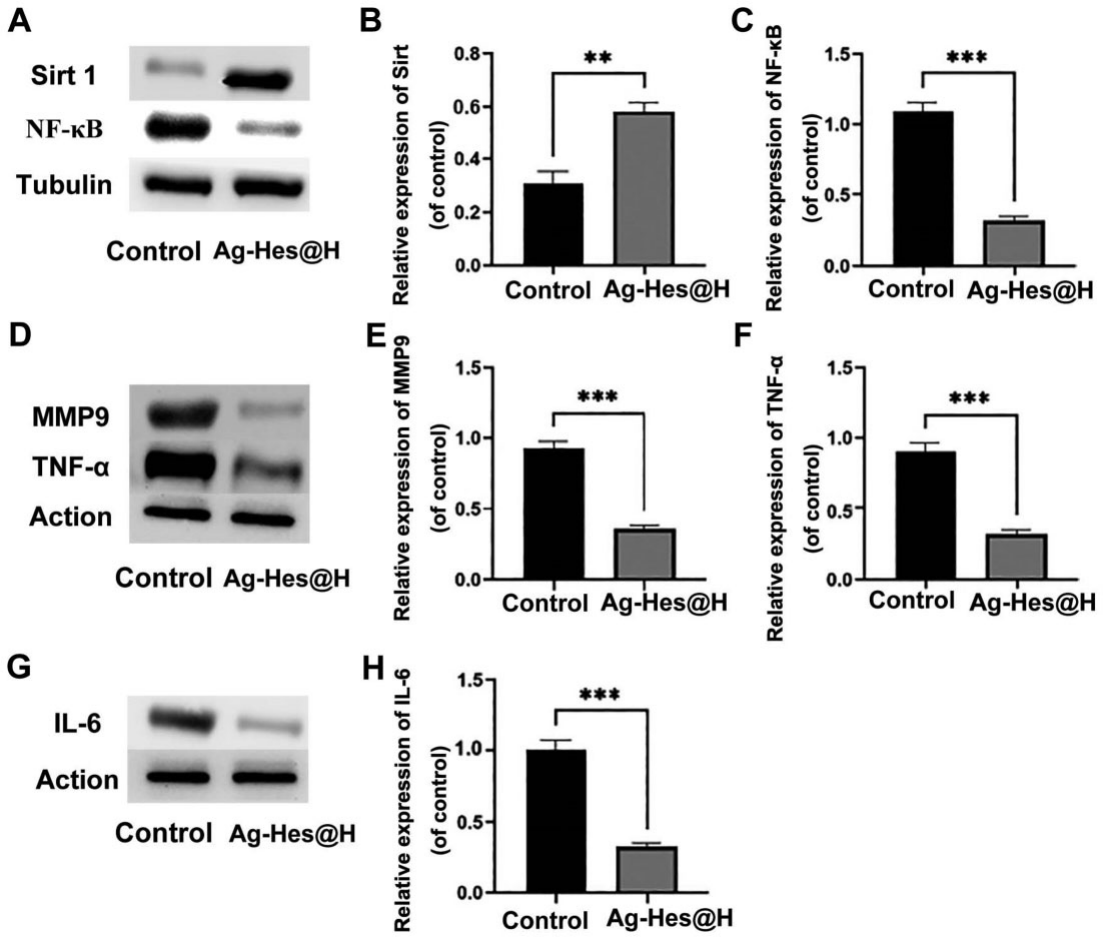
Western blot analysis and quantification of the inflammatory cytokines in wound healing

In order to confirm whether Ag-Hes@H was able to inhibit the production of inflammatory factors, the protein levels of inflammatory factors in wound area were detected by western blotting. NF-κB is widely involved in biological processes such as cell apoptosis, immune response and inflammation [41, 42]. Sirt 1 can regulate inflammation and participate in oxidative stress, directly or indirectly inhibit the activity of NF-κB and reduce the production of IL-6, MMP9, TNF-α and other inflammatory factors, so as to achieve the effect of alleviating neuroinflammation and exerts a neuroprotective effect [43–45]. Figure 10A–C showed that the expression level of Sirt 1 in the Ag-Hes@H group was higher than that of the control group, while the expression level of NF-κB was decreased from control to Ag-Hes@H group. Meanwhile, the expression levels of inflammatory factors MMP9, TNF-α and IL-6 in the Ag-Hes@H group were all suppressed (Fig. 10D–H). Based on the above results, the possible bio-degradation mechanism of Ag-Hes@H nano gel fiber in promoting infected wound healing is schematically illustrated in Supplementary Fig. S7. The gel network gradually degraded after exposure to the physiological environment. Furthermore, Ag-Hes nanoparticles would be released from the degraded gel network. At the same time, polymerized Hes shell might degrade and release Hes fragments and Ag nuclei when contact with reducing substances in the physiological environment, such as GSH. The released Ag core and hesperidin molecules afforded the abilities to inhibit bacterial growth and reduce inflammatory factors for Ag-Heps@H.

## Conclusion

In this work, 20 nm ultra-uniform, colloidally stable Ag-Hes NPs with core-shell structure were prepared. TG result indicted that the amount of Hes incorporated in Ag-Hes NPs was 1.7 wt%. The obtained Ag-Hes NPs present effective antibacterial properties on both *S. aureus* and *E. coli* (inhibition rate > 90%), and reached high DPPH scavenging capability of 69%. Cell experiments and animal wound healing investigation proved that Ag-Hes@H could promote the proliferation and migration of HUVECs cells and accelerate infected wound healing. Meanwhile, Ag-Hes@H significantly reduced the expression of inflammatory cytokines, including IL-6, MMP9 and TNF-α. Immunohistochemistry data further suggested that Ag-Hes@H accelerated wound closure (the wound healing closure rate was close to 97%) by promoting collagen deposition and skin cell proliferation. The designed antibacterial and anti-inflammation Ag-Hes@H has great potential for promoting infected wound healing.

## Supplementary data

Supplementary data are available at *REGBIO* online.

## Funding

This work was supported by National Natural Science Foundation of China (No. 82072076), and 2021 scientific research fund project of Liaoning Provincial Department of Education (LJKZ0819).

*Conflict of interest statement*. The authors declare that the research was conducted in the absence of any conflict of interest.

## Supplementary Material

rbac012_Supplementary_Data
