# Supplementary material for: Electrospinning of antibacterial and anti-inflammatory Ag@hesperidin core-shell nanoparticles into nanofibers used for promoting infected wound healing
Source: Regen Biomater. 2022 Feb 18;9:rbac012. doi: 10.1093/rb/rbac012 (PMC9113224; doi:10.1093/rb/rbac012)
Supplement: rbac012_Supplementary_Data [file rbac012_supplementary_data.docx]

**Supporting information**

**
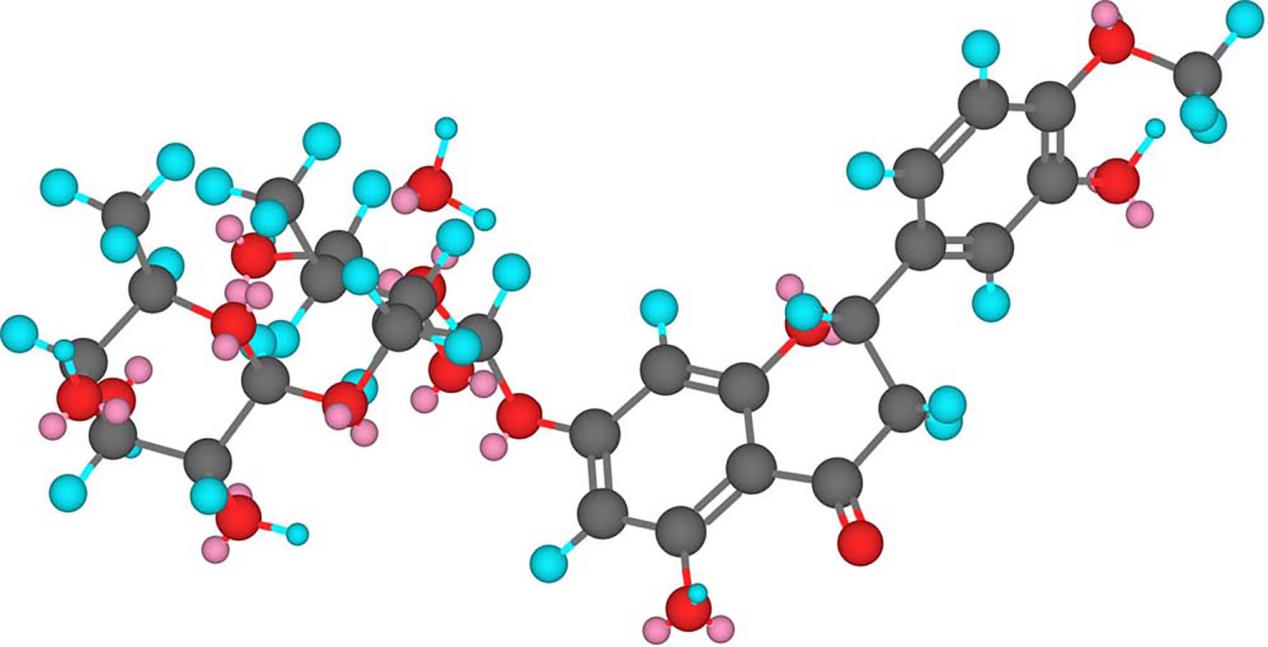
**

**Fig. S1** The Schematic of Hesperidin molecule structure.


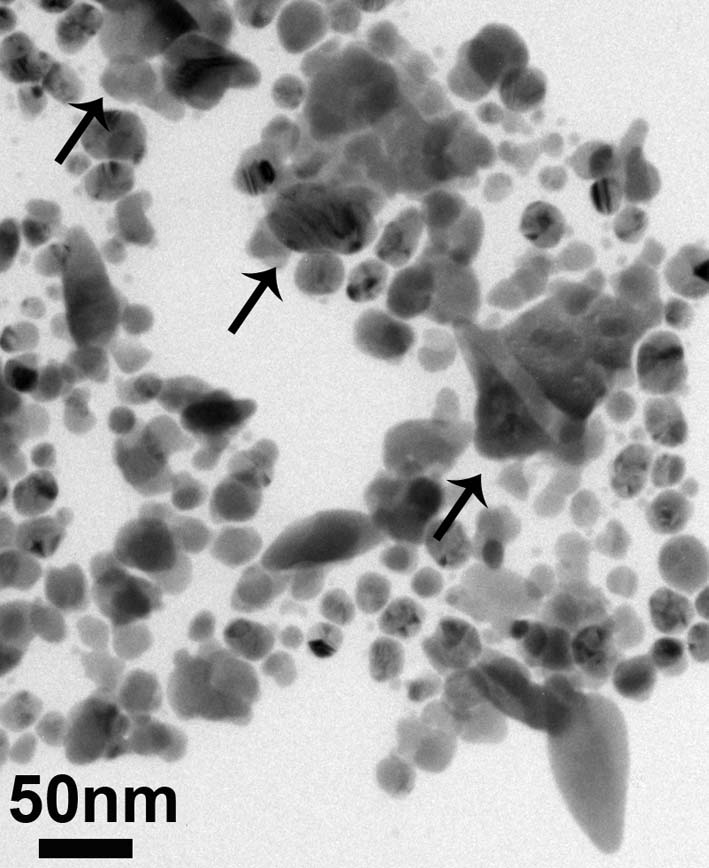


Fig S2 TEM images Ag nanoparticles obtained in the control experiment.


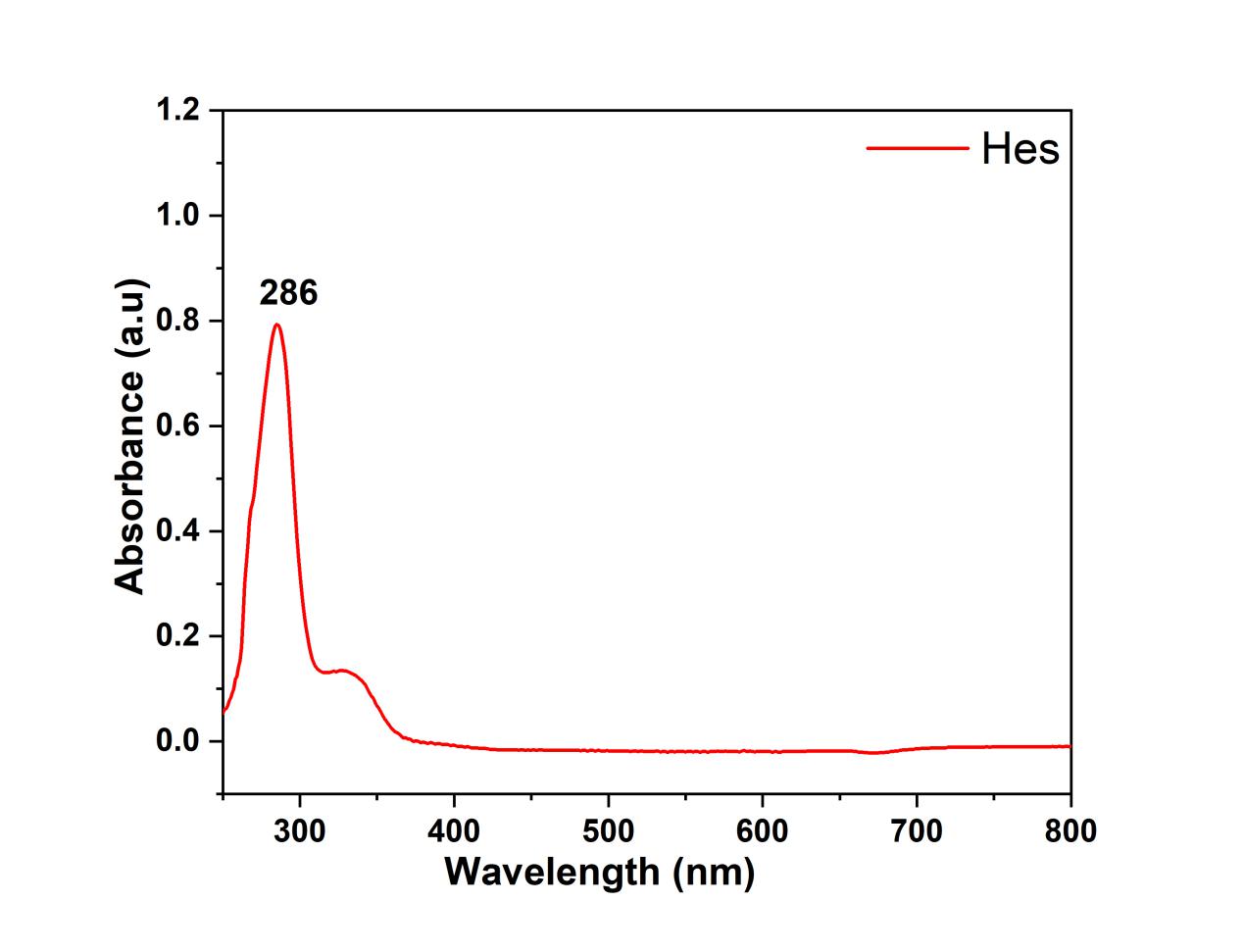


Fig S3 UV-vis spectrum of hesperidin in water.


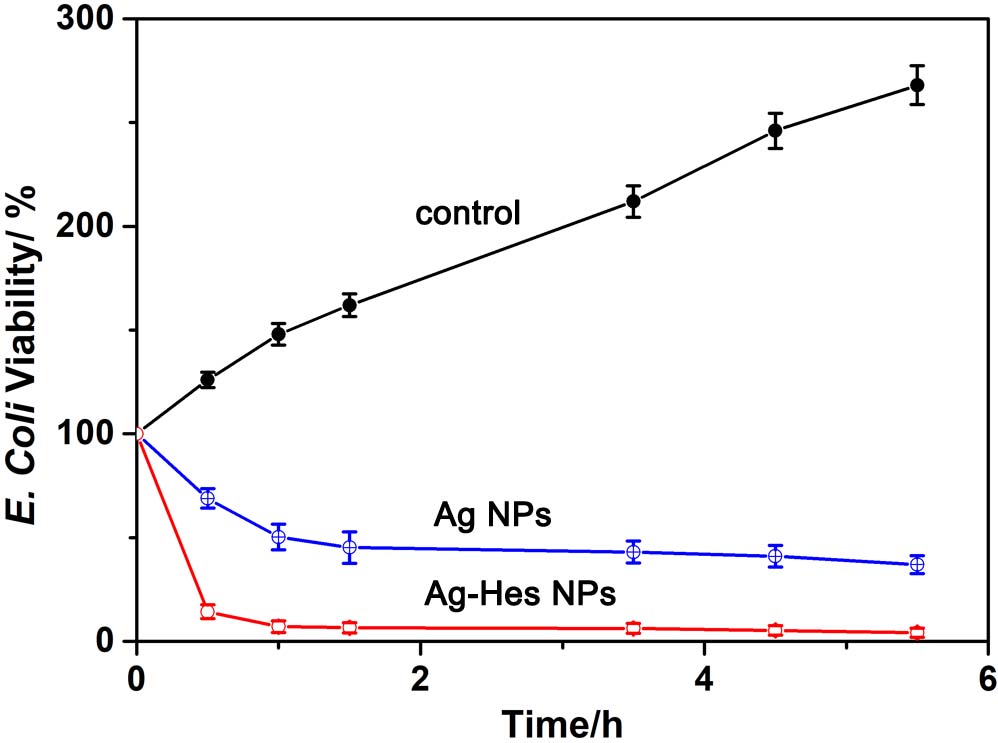


Fig S4 Time-dependent viability curves of *E. coli* with different samples.


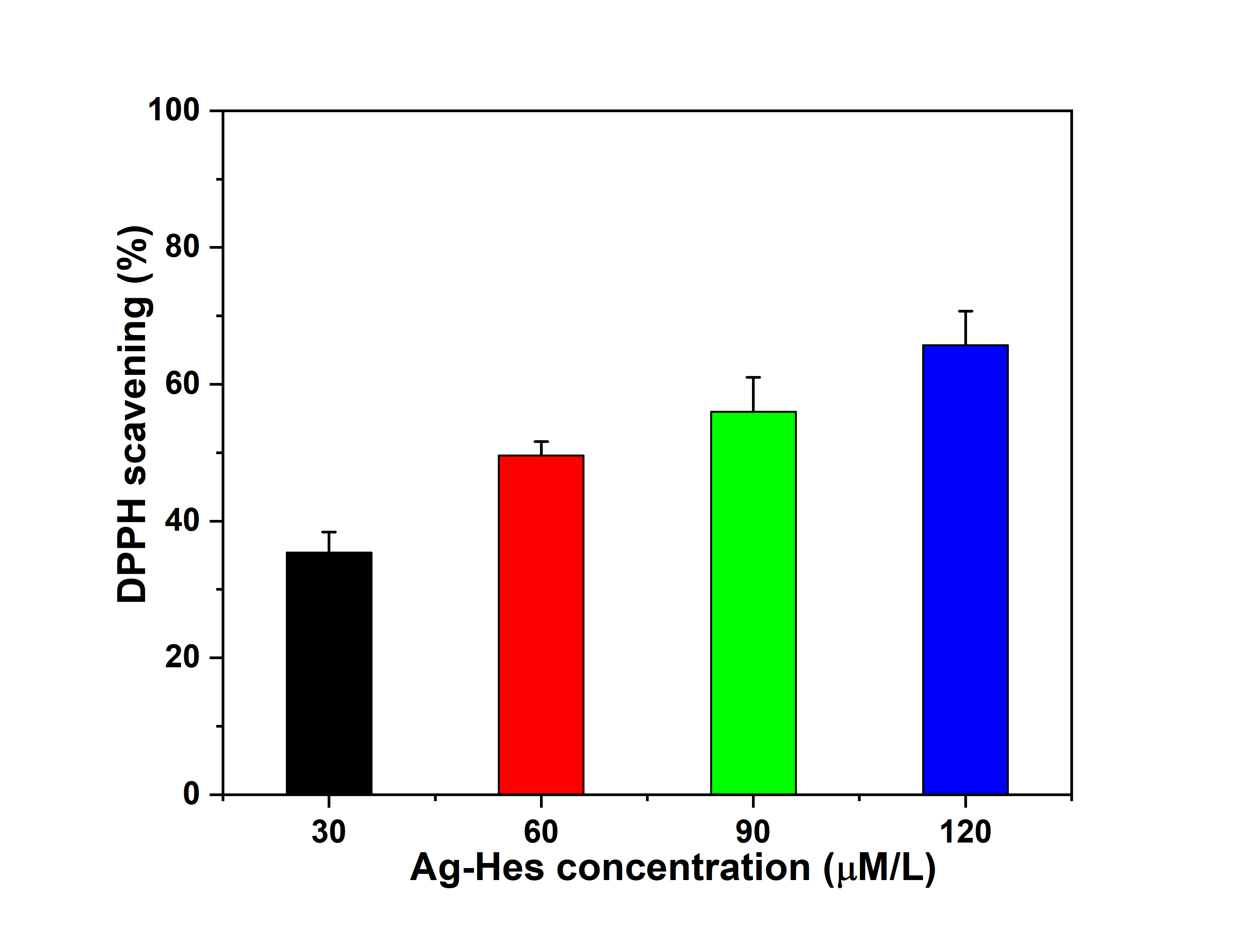


Fig S5 The DPPH scavenging capability of Ag-Hes.


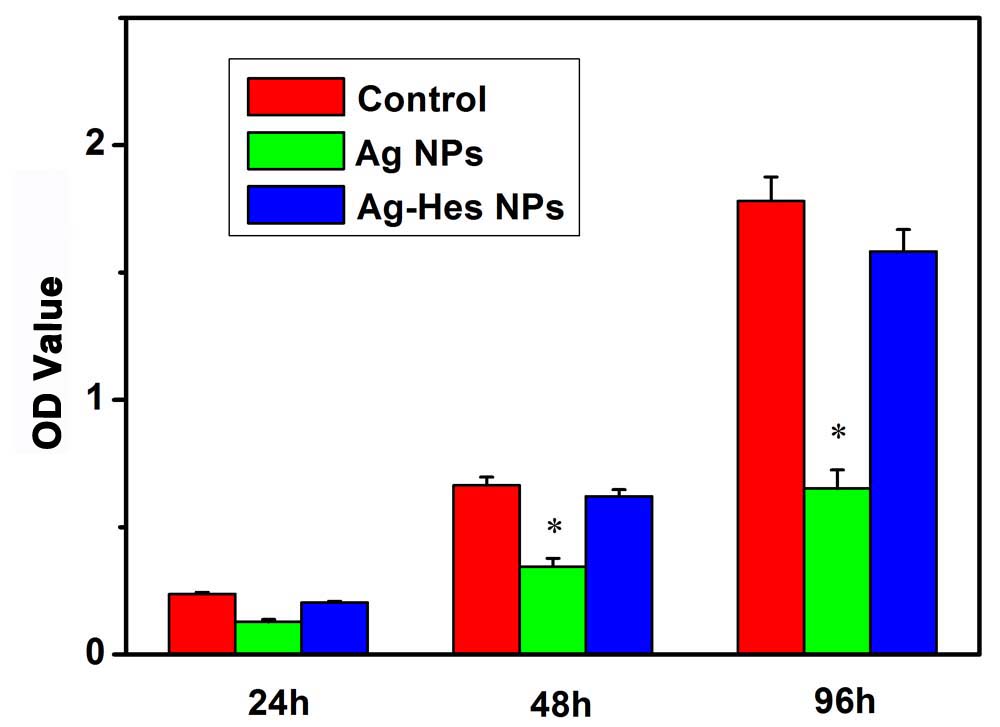


Fig S6 HUVECS cell viability incubated under different conditions at 24, 48 and 96 h.


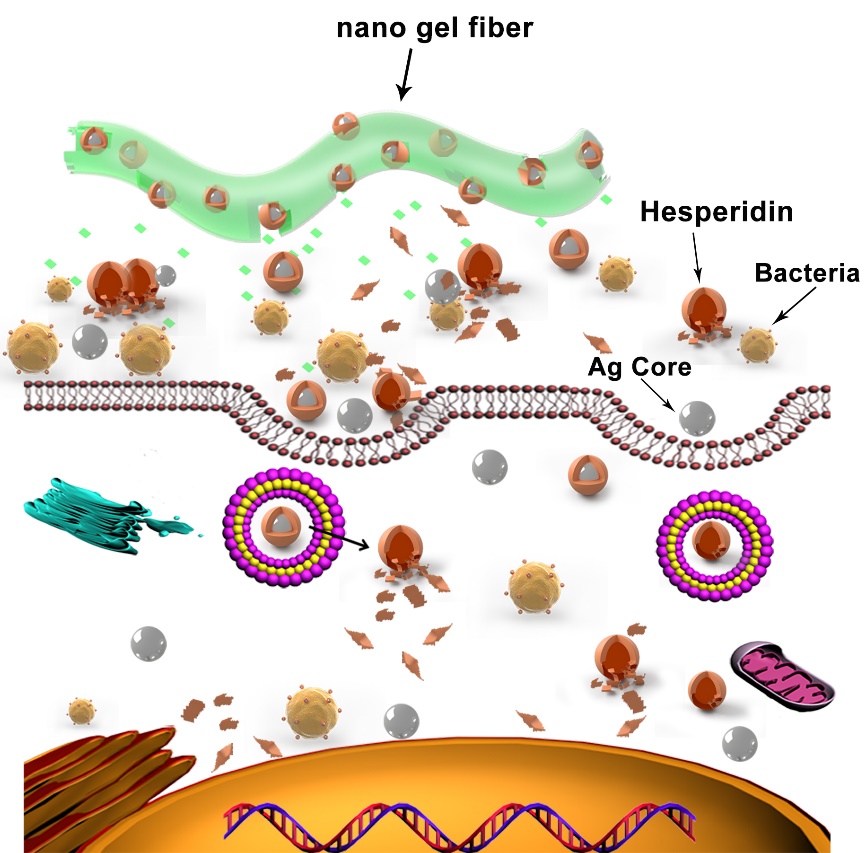


Fig S7 Schematic possible bio-degradation mechanism of Ag-Hes@H nano gel fiber in promoting infected wound healing.
